# Depression and Quality of Life in Patients with Gliomas: A Narrative Review

**DOI:** 10.3390/jcm11164811

**Published:** 2022-08-17

**Authors:** Yue Hu, Fang Deng, Lupeng Zhang, Keyue Hu, Shiqi Liu, Suye Zhong, Jun Yang, Xiaomin Zeng, Xiaoning Peng

**Affiliations:** 1 Department of Clinical Medicine, Hunan Normal University School of Medicine, 371 Tongzipo Road, Changsha 410006, China; 2 Department of Epidemiology and Health Statistics, Xiangya School of Public Health, Central South University, 238 Shangmayuanling Lane, Changsha 410078, China; 3 Department of Biochemistry and Molecular Biology, Jishou University School of Medicine, 120 Renmin South Road, Jishou 416000, China

**Keywords:** glioma, depression, quality of life, mood disorders, antidepressants

## Abstract

In patients with gliomas, depression is a common complication that may cause severe psychological barriers and deteriorate the patient’s quality of life (QoL). Currently, the Hospital Anxiety and Depression Scale (HADS) is the most commonly used tool to diagnose depression in patients with gliomas. Female sex, unmarried status, low education level, high tumor grade, and a history of mental illness may increase the risks of depression and depressive symptoms in patients with gliomas. The QoL of patients with gliomas can be directly reduced by depression. Therefore, the evaluation and intervention of mood disorders could improve the overall QoL of patients with gliomas. Antidepressant use has become a treatment strategy for patients with gliomas and comorbid depression. This narrative review summarizes the current issues related to depression in patients with gliomas, including the prevalence, risk factors, and diagnostic criteria of depression as well as changes in QoL caused by comorbid depression and antidepressant use. The purpose of this review is to guide clinicians to assess the psychological status of patients with gliomas and to provide clinicians and oncologists with a new treatment strategy to improve the prognosis of such patients.

## 1. Introduction

Mental/psychological disorders, including anxiety disorders, mood disorders, alcohol use disorders, cognitive disorders, and dementia, are associated with an increase in other mental disorders and a range of general medical conditions, which lead to reduced quality of life (QoL), increased health-care burdens, and premature death [1,2]. The lifetime and 1-month prevalence of mental disorders were found to be 24.20% and 14.27%, respectively [1]. Anxiety disorders, the most common type of mental disorder, are characterized by excessive fear and anxiety or the avoidance of persistent, harmful perceived threats, and are often comorbid with other mental disorders, especially depression, as well as physical disorders [3]. Mood disorders are characterized by changes in emotion and are presented as unipolar or bipolar depressive episodes [4]. Depression, a psychological disorder characterized by low mood or loss of interest, is a common mental disorder in patients with cancers [5]. The one-year and lifetime prevalence of clinical depression in the general population are 7.2% and 10.8%, respectively [6]. Depression can be an independent risk factor for death, cause severe psychological barriers, deteriorate the patients’ QoL, and even lead to suicide in patients with cancers [5,7].

Glioma is the most common type of primary brain tumor with an annual incidence of approximately six cases per 100,000 people [8,9]. Glioblastoma is the most common tissue type in glioma, accounting for approximately 61% of all gliomas [8]. The 2-year survival rate after diagnosis is only 27%, and age and resection range are important factors affecting the prognosis of patients with gliomas [8,10]. Symptoms of glioma include seizures, focal neurological symptoms, personality changes, mood disturbances, and depressive symptoms, with depression being a major complication of primary glioma [11,12]. In a prospective observational cohort study involving 155 patients with gliomas, one in five patients developed clinical depression after the diagnosis of glioma, with major depressive symptoms lasting at least three months [13].

Psychological distress and cognitive impairment are closely related to poor QoL and prognosis in patients with gliomas. In particular, depression and anxiety can significantly reduce patients’ QoL and even lead to suicide. Therefore, clinicians highly recommend assessing the relationship between psychological distress, subjective cognitive complaints, and neuropsychological performance in patients with gliomas [14,15].

## 2. Prevalence of Mental Disorders in Glioma

Mental disorders are common in patients with cancers, with high levels of emotional distress and psychosocial stress [16,17,18,19]. A multicenter epidemiological study assessed the 12-month and lifetime prevalence of all mental disorders in patients with cancers [16]. Kuhnt et al. [16] consecutively recruited patients with cancers, including major tumor entities and stages, from acute-care hospitals, cancer outpatient care facilities, and cancer rehabilitation clinics and screened eligible patients using the depression module of the Patient Health Questionnaire-9 (PHQ-9). After applying exclusion criteria, such as refusal to provide informed consent and loss to follow-up, 2142 patients were analyzed [16]. The prevalence of mental disorders within 12 months of cancer diagnosis was 39.4%, and the most common psychiatric disturbances were anxiety disorders (15.8%) and mood disorders (12.5%). The lifetime prevalence of mental disorders was 56.3%, and the most common psychiatric disturbances were anxiety disorders (24.1%) and mood disorders (20.5%). Patients with head and neck cancer had the highest lifetime prevalence of axis I mental disorders (mainly anxiety and mood disturbances), diagnosed in 69.9% of patients [16].

Mental disorders, mainly including depression and anxiety, are common in patients with gliomas [20,21,22,23,24,25]. In a recent retrospective study, the prevalence of preoperative depression, anxiety, and cognitive impairment in patients with gliomas was 53.5%, 70.4%, and 32.4%, respectively [20]. Regarding the severity of mental disorders, moderate to severe depression and anxiety were diagnosed in 28% and 36% of patients with gliomas, respectively [21]. However, on different assessment scales, the prevalence of depression in patients with gliomas ranged from 28.4% to 32.6% [22]. Notably, Wang et al. [23] and Piil et al. [24] found that the incidence of mental disorders was related to the severity of glioma. Among patients with high-grade gliomas, 46% had depression-related symptoms [23], while 26.7% were diagnosed with depression [24]. In a prospective cohort study involving 155 patients with gliomas followed up for six months, 20.6% of patients were diagnosed with major depressive disorder (MDD) in the study period, with MDD symptoms persisting for at least three months [13]. In addition, patient age is associated with psychological distress in patients with gliomas. Young patients with malignant gliomas tend to have higher levels of anxiety during surgery and radiotherapy, suggesting that young age may be a potential risk factor for anxiety in postoperative patients with gliomas [25]. Table 1 summarizes major studies on the prevalence of psychiatric disorders —primarily depressive disorders—in gliomas and the results of major studies on the association between depression and prognosis in patients with gliomas.

## 3. Diagnostic Criteria for Depression in Patients with Gliomas

Diagnosing depression in patients with cancers is difficult, because the tumor itself (e.g., size and location) or treatment (e.g., surgery, chemotherapy, and radiotherapy) may directly or indirectly contribute to depression-related symptoms. For example, in the Diagnostic and Statistical Manual (DSM) of Mental Disorders criteria for diagnosing MMD [26], symptoms include changes in appetite, sleep status, fatigue, and decreased concentration, which may be caused by the tumor itself or surgery-related treatment. Evidence supporting the hypothesis that the tumor or surgery is the direct cause of comorbid depression in patients with gliomas is lacking [27]. Depression is also difficult to distinguish from normal psychological sadness in patients with advanced cancers. Identifying hopelessness, anhedonia, guilt, and suicidal ideation is an effective way to distinguish depressive symptoms from general sadness [27,28]. Clinicians may dismiss depressive symptoms and assume that sadness is normal in patients diagnosed with gliomas; therefore, more rigorous and precise scales or methods should be developed for clinicians to assess depressive symptoms in patients with gliomas [27,28].

Currently, depression is clinically diagnosed using depression scales, such as the Beck Depression Inventory (BDI), Hospital Anxiety and Depression Scale (HADS), Center for Epidemiologic Studies Depression Scale (CES-D), PHQ-9, and self-rating scales, of which HADS is the most commonly used tool [11,27,29]. Several depression screening tools, such as the Schedule for Affective Disorders and Schizophrenia, BDI, HADS, and CES-D, have been validated to screen for depression in patients with cancers with relative readiness [30]. Some studies reported that the depression subscale of HADS, with a lower threshold of 8, and PHQ-9, with a threshold of 10, are more effective and sensitive tools for the screening of clinical depression symptoms in patients with gliomas [31]. Others reported that face-to-face interviews are the best method for the clinical diagnosis of depression in patients with gliomas [27]. Rooney et al. [11], in an observational study, screened 42 studies on depression involving adults with primary gliomas. BDI (median = 39%, range = 38–42%) had a higher frequency of screening depression compared to HADS (median = 16%, range = 0–21%). The median frequency of glioma-related depression in clinical interviews was 15% (range = 6–28%). Patient-scoring methods generally showed a higher prevalence of depression (median = 27%; range = 0–93%) compared to clinician-scoring methods (median = 15%; range = 5–28%) [11]. HADS was recommended as an initial screening tool for depression rather than BDI, which could amplify the frequency of depression compared to clinical interviews [11].

However, no depression screening tool has been validated to assess depressive symptoms in patients with gliomas [31]. Possible explanations are as follows: (1) the infiltrative nature of gliomas, destructive surgery, and the site of radiation therapy often being the site of depression; (2) the lack of expertise of clinicians and oncologists in the assessment of depressive symptoms; (3) qualitative differences between gliomas and other cancers; and (4) errors and reduced validity of self-reports induced by fatigue and cognitive impairment. Multiple confounding factors influence the judgment of depressive symptoms in patients with gliomas; therefore, findings from depression screening tools validated in patients with other cancers often cannot be applied to patients with gliomas [31,32,33]. In addition to focusing on characteristic symptoms of depression in patients with gliomas, such as hopelessness, anhedonia, and suicidal ideation, attention should be paid to functional impairment, a history of depression, and female sex, which are considered to be possible risk factors for depression in patients with gliomas [11,34].

## 4. Current Knowledge

In this narrative review, we summarize the current knowledge about depression in patients with gliomas and answer the following questions:

A. What are the risk factors for depression in patients with gliomas?
B. Is depression a risk factor for the development of glioma?
C. Is depression a prognostic factor for mortality in glioma?
D. Is glioma a risk factor for depression?
E. Is there a correlation between depression and glioma severity?
F. How does depression affect the QoL of patients with gliomas?
G. What is the relationship between antidepressants and the risk and prognosis of glioma?

## 5. Depression and Risk Factors in Glioma

Study findings are inconsistent for the risk factors of depression in patients with gliomas. Depression in patients with gliomas may be related to the following factors: gender, education level, World Health Organization (WHO) tumor grade, psychiatric history, marital status, and current/past medical history [22,33,34,35]. Some studies suggested that female sex, unmarried status, low education level, high tumor grade, and history of mental illness were associated with increased risks of depression and depressive symptoms in patients with gliomas [22,33,35]. However, another study concluded that female sex, marital status, educational level, WHO tumor grade, chemotherapy, radiotherapy, and psychiatric history were not associated with depression in patients with gliomas [11].

### 5.1. Gender

In the general population, women show a higher risk for depression [36,37]. Arnold et al. [35], through a questionnaire survey and demographic data analysis of 363 adult patients with gliomas, reported that 60% of women with gliomas had depression or anxiety symptoms, indicating that female sex was a risk factor for depression, both in the general population and in patients with gliomas. This phenomenon has been explained by various hypotheses, including genetic, psychosocial, and diverse physiological factors [35,36,37,38]. Temporal changes in estrogen levels may affect women’s emotional and psychological state, leading to a higher tendency towards depressive symptoms in psychologically vulnerable women [38]. In a previous study, approximately 40–44% of women with depression and 21–31% of men with depression had a genetic component, suggesting that women with a family history of depression may be more prone to depression [39]. A clinical study found that female caregivers of patients with gliomas were more likely to have preoperative psychological distress, including depression (*p* = 0.008). The study also found that the preoperative depression of patients with gliomas was positively correlated with the depression of caregivers, which may be because glioma is a highly disabling disease and mainly depends on the support of caregivers [40]. However, the study did not analyze the effect of gender on the development of depression in patients with gliomas. In a prospective study, 190 patients with gliomas were assessed for depression using HADS and SDS. This study found that female sex was an independent risk factor for SDS depression in patients with gliomas, while gender was not associated with HADS depression [22]. However, when Rooney et al. [41] applied a depression questionnaire to 100 patients with gliomas (55 men and 45 women) from the Edinburgh Hospital database [41], both sexes had a similar risk for depressive symptoms after the diagnosis of glioma (12/55 and 12/45, respectively; *p* = 0.64), whereas women had a significantly higher risk compared to men before the diagnosis of glioma (12/45 and 3/55, respectively; *p* = 0.004). Thus, after the diagnosis of glioma, the gender difference in depression between men and women disappeared, i.e., female sex was not a risk factor for depression in patients with gliomas [41]. Similarly, in an observational study by Rooney et al. [11] in 2011, female sex was not a significant risk factor for depression in patients with gliomas, and this absence of sex predilection might indicate an increased relative risk of depression in men with gliomas.

### 5.2. Marital Status

In a study by Arnold et al. [35], unmarried patients were more likely to develop depression compared to married patients, although without statistical significance. Similarly, in a study on prognostic indicators of head and neck cancer, married patients had a lower prevalence of psychiatric symptoms, which may be related to the marital status affecting the prognosis through mechanisms of healthy behavior and/or social support, and a low depression level in married patients was as an independent predictor of the prognosis of glioma [42]. The prospective study of Hao et al. [22] used different depression scales to evaluate patients with gliomas and found that in single/divorced/widowed patients, relationship status was an independent risk factor for HADS and SDS depression. The researchers suggested that this might be because single/divorced/widowed patients received less social support and were therefore more prone to depression [22]. However, in the observational study by Rooney et al. [11] including six studies on the relationship between the marital status and depression, marital status (married or unmarried) was not a risk factor for depression in patients with gliomas.

### 5.3. Education

The education level of patients with cancers is significantly correlated with depressive symptoms (r = −0.785, *p* < 0.001), and high levels of social support correspond to low levels of depression. Patients who receive support from family members and government aid have lower levels of depressive symptoms. The educational level of patients with cancers is significantly associated with depressive symptoms (*p* = 0.04) [43]. This may be related to the greater healthcare needs of people with higher education levels. People with higher education levels may have a higher income, enabling them to use medical resources and receive follow-up treatments [35,44]. Arnold et al. [35] could not establish whether the high prevalence of depression in patients with low education levels was associated with socioeconomic issues, such as financial stress or health insurance. Furthermore, they could not explain the more severe depression tendencies in patients who had completed college [35]. Hao et al. [22] also found that a duration of education < 12 years was an independent risk factor for HADS depression in patients with gliomas, while education level was not associated with SDS depression. This could be because patients with low levels of education had less knowledge of gliomas and were more likely to panic, which increased the incidence of depression [22]. Rooney et al. [11] analyzed three studies using different definitions of education to investigate the relationship between the education level and glioma-related depression and concluded that the education level was not a risk factor for depression in patients with gliomas.

### 5.4. Tumor

No evidence suggests that the WHO tumor grade, tumor location, or tumor size is associated with depression in patients with gliomas [11,22,45,46,47]. Although, in previous studies, low-grade neuronal tumors were significantly associated with neuropsychiatric disorders, this trend was statistically significant only in anxiety disorders [35]. In a longitudinal multicenter study involving 589 patients with high-grade gliomas, patients with multifocal and large tumors were more likely to experience postoperative depression-related symptoms than those with unifocal or small tumors [45]. However, this study only included patients with high-grade gliomas in the postoperative period and did not investigate depression in patients with low-grade gliomas or in the pre- or intra-operative period [45]. Hao et al. [22] found that WHO grade was associated with anxiety in patients with gliomas, but did not find an association between WHO grade and depression in patients with gliomas [22]. The previous observational study, which included 42 studies, concluded that no studies had examined the association of different tumor locations with the development of depression [11]. In 2017, a prospective cohort study found that tumor location was not associated with HADS scores (including scores for depression) in patients with gliomas (*p >* 0.05) [21]. Later, a study involving 77 patients with low-grade gliomas found that a tumor in either hemisphere affected cognitive function and that there was no significant difference in cognitive function scores between the two hemispheres [46]. Therefore, further research is required on the effect of the tumor size and location on depressive symptoms in patients with gliomas.

### 5.5. Treatment

Evidence suggests that radiation or chemotherapy has no direct effect on depressive symptoms in patients with gliomas [11,13]. However, in a recent prospective study, chemotherapy and radiotherapy caused depression in patients with gliomas, particularly after 3 months. Effective measures should be undertaken to intervene in depressive symptoms in patients with gliomas within 3 months of the completion of radiotherapy or chemotherapy [47]. Litofsky et al. [45] proposed that glucocorticoids may be associated with depression. However, side effects of glucocorticoid use, including fatigue, sleep disturbance, and cognitive dysfunction, overlap with depressive symptoms [48]. Corticosteroid use may increase the risk of depression in patients with gliomas, despite conflicting evidence [13]. In addition, no evidence suggests that antiepileptics may be associated with depression in patients with gliomas [11].

### 5.6. Psychiatric History

Rooney et al. [11] analyzed four studies on the relationship between psychiatric history and depression and found that psychiatric history was not a risk factor for depression in patients with gliomas. Arnold et al. [35] reported that many patients with a history of mental illness were diagnosed with an intracranial tumor. They hypothesized that the pathophysiological process or treatment of brain tumors may affect the development of depression and anxiety. However, further research is required to test this hypothesis [35]. In a study by Hao et al. [22] evaluating depression in 190 consecutive patients with gliomas, HADS revealed that education < 12 years, female sex, marital status (single/divorced/widowed/married), and chronic kidney disease were independent risk factors for depression, while the Zung self-rating depression scale revealed that female sex, marital status (single/divorced/widowed/married), and hyperlipidemia were independent risk factors for depression [22]. In a study involving 363 patients with gliomas (response rate, 66%) and 481 cancer-free patients (response rate, 37%) diagnosed within 12 months at neurosurgery clinics in Denmark, the prevalence of depression did not differ significantly by sex, age, tumor grade, marital status, or education level, inconsistent with previous findings [49]. The study was limited in that antidepressant use in the follow-up period was not investigated, possibly underestimating the prevalence of moderate-to-severe depression, and patients with significant cognitive impairment were excluded, possibly deceasing the generalizability of the results to the entire population with depression [49].

Although study results on the risk factors of depression in patients with gliomas are contradictory, sex, marital status, tumor grade, and education level could be potential risk factors.

## 6. Depression as a Risk Factor for Glioma

The potential link between psychosocial factors and cancer incidence and progression has attracted great interest from the scientific community and the public [50]. In a meta-analysis of 46 studies, specific psychosocial factors, i.e., depression and anxiety, were strongly associated with breast cancer [51]. In addition, the development of gliomas has been reported in patients with depression [52,53]. In an observational study, lifestyle factors, such as chronic stress, had a significant direct link with the development of brain tumors [54]. In a retrospective case–control study by Peeters et al. [55] in 2019, involving 14 studies on pre-diagnostic conditions in patients with gliomas, eight conditions had a higher incidence before the diagnosis, including epilepsy, dyskinesia, confusion, language problems, memory problems, personality changes, altered consciousness, and visual problems, of which the most prevalent was mood disturbance [55]. When patients with undiagnosed glioma see a general practitioner with a mood disorder, the general practitioner would not consider glioma as a diagnosis because mood disorders cannot be used to distinguish patients with gliomas from those with other neurological disorders. Therefore, the study suggested that general practitioners should consider all central nervous system disorders, including glioma, when mood disorders are present [55].

Depression and glioma are associated with epidemiological factors and clinical and pathological features. A common neurological manifestation in patients with depression is the hyperfunctioning of the hypothalamic–pituitary–adrenal axis, leading to elevated secretion of the corticotropin-releasing factor [56,57]. However, elevated secretion of the corticotropin-releasing factor could inhibit the apoptosis of some glioma cells [58]. Palma et al. [50] mentioned the role of tachykinin in depression and glioma. Tachykinin, a neuromodulator involved in regulating physiological functions, could be involved in the pathogenesis of many diseases, such as the occurrence and development of depression [50]. It can act at different stages of the carcinogenesis of glioma and neuroblastoma, affecting signal transduction in normal cells, promoting the proliferation and survival of cancer cells, and releasing cytokines and soluble mediators that favor tumor growth [50]. The involvement of astrocytes in depression has been reported, but valuable studies on the relationship between the genetic component of depression and glioma development and progression are still lacking [59,60,61]. Coppola et al. [59] used bioinformatics analysis to further delineate the key mediators involved in the astrocyte–peripheral neural nets–depression relationship and identified important transcription factors in astrocytes [59,60]. Fibroblast growth factors (FGFs) and their receptors have been shown to be involved in the pathogenesis of neurological diseases. FGF2 may indirectly enhance neuronal activity by stimulating astrocyte proliferation, which is decreased in rodent models of depression [61].

Although the development of glioma in patients with depression has been reported, studies on pre-diagnostic symptoms of patients with gliomas, particularly depression, are few. Further research is required to clarify whether depression can be a predictor of the development of glioma.

## 7. Depression as a Prognostic Factor for Glioma Mortality

A study established depression as an independent predictor of mortality in patients with cancers outside of the central nervous system [7]. Further, depression was found to be an independent predictor of mortality in brain tumor patients [62]. However, whether or not depression is a prognostic factor for mortality in patients with gliomas remains uncertain.

Comorbid depression in patients with gliomas is associated with functional impairment, cognitive impairment, and reduced QoL [13], and depression is associated with mortality in patients with gliomas [45,63,64]. In a retrospective study on the relationship between preoperative depression and postoperative survival, depression was a predictor of reduced survival time in patients with high-grade gliomas, independent of disability, tumor grade, or subsequent treatment [63]. In a meta-analysis by Shi et al. [29], depression was associated with poor survival outcomes, irrespective of the duration of depression, particularly in patients with high-grade gliomas [29]. In another cohort study, depression was a predictor of poor outcomes and associated with decreased survival in patients with high-grade gliomas. Moreover, patients with gliomas and combined depression had more complications within six months postoperatively, such as deep vein thrombosis, epilepsy, infection, and adverse drug reactions [45]. In an analysis of patients with low-grade gliomas, depression was a potential prognosticator for poor survival in patients with low-grade gliomas, with depressed patients having significantly shorter 5-year survival compared to non-depressed patients [64]. Patients with gliomas and combined depression may have poor neurological function and performance status, be prone to more complications, and experience impaired interpersonal relationships, which may be related to their decreased survival [13,45]. However, in a study by Bunevicius et al. [21], depression or anxiety symptoms were not associated with mortality in patients with gliomas.

## 8. Glioma as a Risk Factor for Depression

Many studies have confirmed that patients with gliomas are more likely to suffer from depression compared to the general population [13,64]. Patients with intracranial tumors are at a higher risk for depression [64]. In a previous review of 42 observational studies on depression in patients with gliomas, the median prevalence of depression was 27% [11]. In the largest serial cohort study to date, involving 155 patients, one in five patients developed clinical depression six months after the diagnosis of glioma [8]. The one-year and lifetime prevalence of depression in the general population are 7.2% and 10.8%, respectively [6]. Glioma compresses brain tissue and presents with a range of neurological deficits, including personality changes, aphasia, apathy, auditory or visual hallucinations, mania, panic attacks, and amnesia, which are similar to depressive symptoms and thus enable an easy diagnosis of depression [65]. No evidence suggests that the WHO tumor grade, tumor location, or tumor size is associated with depression in patients with gliomas [11,21,22,45,46].

## 9. Depression and Glioma Severity

Some current findings suggest that patients with high-grade gliomas are more likely to be diagnosed with depression [23,66]. In other words, depression may be associated with glioma severity. However, this finding remains controversial.

Clinical depression is a common complication of high-grade glioma [62]. In previous studies, among all patients with cancers, those diagnosed with high-grade glioma had the highest risk for clinically significant psychiatric complications after their cancer diagnosis [62,67]. In a study by Wang et al. [23], approximately 46% of patients with high-grade gliomas developed depressive symptoms. In a study by Fox et al. [66], 73 patients with high-grade gliomas had a depression rate of up to 95% after the glioma diagnosis, probably because most of the patients with high-grade gliomas had behavioral, emotional, and intellectual difficulties, impairing their ability to live independently and perform daily tasks and other activities, placing additional stress on both the patients and caregivers [23,66]. In addition, depression may also be a psychological response to catastrophic changes and a threat to life [23,66]. In a study by Piil et al. [24], the prevalence of depression in patients with high-grade gliomas was 26.7%, different from the prevalence of depression in patients with gliomas according to different assessment scales (28.4%–32.6%), and depression had no correlation with glioma severity [22].

## 10. Depression and QoL in Glioma

QoL has no unified definition internationally and is generally affected by health. Health-related quality of life (HRQoL) is a more accurate term compared to “QoL”, which is a complex multidimensional structure and includes a series of conceptual definitions. HRQoL encompasses a complex set of factors covering general health, physical symptoms, cognitive issues, well-being, role functioning, social functioning, job satisfaction, and finances. Factors related to QoL are important for patients and their caregivers, and the HRQoL of patients with cancers has been extensively studied [68,69]. The QoL of patients with cancers may be affected by depression. In a large cohort study, involving 57,621 patients with malignant intracranial tumors, the incidence of comorbid MDD (11.32%) was clinically significant, and depression was consistently associated with functional impairment, cognitive function, directly reduced QoL, and poor perioperative outcomes [70].

Brain tumors may induce intrinsic neurodegenerative processes and directly lead to focal brain dysfunction [71]. Gliomas are highly aggressive and infiltrate the brain parenchyma [72]. Patients with gliomas often present with headache, paralysis, loss of sensation, fatigue, cognitive impairment, anxiety, and depression, which greatly affect the QoL [73]. The current standard of care is aggressive combination therapy, including surgical resection, adjuvant radiotherapy, and chemotherapy. However, even with maximal therapy, the 5-year survival rate is < 5%; therefore, the present treatment goal is to maximize QoL [70]. Many studies have highlighted the necessity to consider QoL issues in the treatment of gliomas [74,75]. In a prospective multicenter study (*n* = 87), patients with gliomas treated with chemotherapy had higher levels of depression, higher chemotherapy-induced nausea and vomiting, and lower QoL at all time points. Therefore, the researchers suggested that depressive symptoms in patients with cancers should be recognized and treated to avoid the more serious side effects of cancer treatment [75]. QoL encompasses symptom control; functional status, such as the ability to carry out activities of daily living; emotional health, such as the control of depression and anxiety; and social health, including the possibility of maintaining personal, family, and social roles and gaining access to social support [76].

Scales commonly used to assess HRQoL in patients with brain tumors have not been comparatively analyzed. Assessment tools for HRQoL have evolved from unidimensional, general questionnaires to multidimensional, specific questionnaires, such as the Karnofsky Performance Status Scale (KPS), Folstein’s Mini-Mental State Examination (MMSE), European Organization for Research and Treatment of Cancer Quality of Life Questionnaire-30 (EORTC QLQ-C30), Brain Cancer Module (BCM), Functional Assessment of Cancer Therapy—Brain (FACT-Br), and Linear Analog Self-Assessment (LASA) scale [68,69]. KPS and MMSE are simpler scales. KPS is widely used to measure the physical function of patients in clinical practice; however, it is a report submitted by doctors or senior nursing staff and cannot directly reflect the QoL of patients. MMSE is commonly used to screen dementia and cognitive impairment, but its sensitivity and specificity are challenged by an inability to adequately assess mild aphasia and agnosia. QLQ-C30, BCM, FACT-Br, and LASA are complex multidimensional scales assessing HRQoL [68]. EORTC QLQ-C30 version 3.0, the most commonly used HRQoL tool in cancer trials, contains 30 items to measure general HRQoL in patients with cancers [69]. BCM and FACT-Br may only be suitable for evaluating HRQoL in high-functioning patients with brain tumors. Patients with a KPS score < 50 are often unable to complete the questionnaires. LASA has been widely used in clinical studies. It is effective and reliable in patients with neurological tumors [68]. Depression negatively affects HRQoL and is considered a major predictor of HRQoL deterioration and survival in patients with brain tumors [68,69]. This may be related to the fact that psychological problems can aggravate cognitive dysfunction, such as tumor location in the left hemisphere; disease progression; and treatment, including radiotherapy, chemotherapy, antiepileptics, and steroids, which can impair cognitive function [77]. A diagnosis of cancer could also induce feelings of fear and depression to a greater extent than other diseases, and depression could hinder psychological growth and the positive effects of coping strategies, thus reducing QoL [69,78].

In a study by Lucchiari et al. [76] evaluating the mental health and QoL of 73 patients with high-grade gliomas, self-perceived scores of QoL significantly differed among patients with and without moderate to severe depression, with the patients with mild depression reporting better QoL. Regarding sex differences, QoL scores were more strongly correlated with psychological distress, such as depression or anxiety, than with KPS in women but strongly associated with KPS in men [76]. In a prospective study on the moderating effect of depression on the relationship between posttraumatic growth and QoL in patients with low-grade gliomas, depression significantly modulated posttraumatic growth and QoL. Compared to other variables, depression and complications negatively affected QoL, suggesting the importance of depression in improving QoL in patients with gliomas [78]. Evidence suggested that depressive symptoms and neurocognitive impairment, particularly executive function, were independently associated with shorter survival in patients with gliomas, and patients with gliomas with both symptoms had the worst prognosis [79]. Outcomes could be improved through psychological assessment, and QoL could be improved through psychological and cognitive interventions [79].

In a study by Noll et al. [80], scores for all subscales of the Functional Assessment of Cancer Therapy Scale, including General Wellbeing, Emotional Wellbeing, Functional Wellbeing, and the brain module, were strongly correlated with depression scores. In other words, depressive symptoms were closely related to the reduction in various aspects of HRQoL. More than half of the patients reported reduced wellbeing [80]. A prospective study of 80 patients with low-grade gliomas and 65 patients with high-grade gliomas found a very high incidence of mood disorders and low HRQoL in the first 3 months after surgery and among patients receiving adjuvant therapy. Since mood disorders and HRQoL had significant intrinsic prognostic value and the improvement of patient health in turn increased the overall survival, the researchers suggested that the evaluation of mood disorders and HRQoL as primary or secondary endpoints must be conducted systematically in patients with gliomas [81]. Therefore, for patients experiencing decreased HRQoL after the diagnosis of glioma and before any treatment, mood disorders should not be ignored or treated alone [66,81].

A nationwide randomized controlled trial of an online guided self-help intervention on depressive symptoms showed that the intervention did not reduce depressive symptoms or improve HRQoL in adults with gliomas. Although effective, it positively affected fatigue, suggesting that further research is required to explore the possible effective strategies to improve depressive symptoms in patients with gliomas [82]. In a study by Wang et al. [23], patients with high-grade gliomas with depression who received psychological intervention had better survival outcomes than those who did not [23]. Based on the current research on the relationship between depression and QoL in patients with gliomas, the evaluation and intervention of mood disorders could improve the overall QoL.

## 11. Antidepressants and Glioma

Antidepressants are widely used in patients with cancers [83]. They reduce depressive symptoms in patients with cancers, but the mechanism is unclear [84]. The incidence of depression in patients with gliomas is high, and antidepressant use has become a treatment strategy for patients with gliomas and comorbid depression. Table 2 summarizes the relevant articles on the relationship between antidepressants and the occurrence and prognosis of gliomas [85,86,87,88,89].

**Figure 1:**
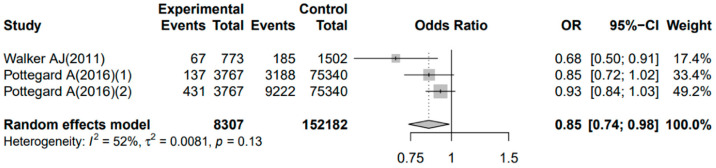
The association between antidepressants and the occurrence of glioma in the three case-control studies [85,86]. OR, odds ratio; CI, confidence interval.

Some studies investigated the effect of antidepressant use on the risk of glioma [85,86]. Tricyclic antidepressants (TCAs) reduce the risk of glioma (odds ratio [OR] = 0.59; 95% confidence interval [CI]: 0.42–0.81), and this effect is the strongest at high doses and with prolonged treatment [85]. However, in a study by Pottegård et al. [86], long-term TCA use was not associated with glioma risk (OR = 0.89; 95% CI: 0.75–1.06). Similarly, long-term selective serotonin reuptake inhibitor (SSRI) use was not associated with glioma risk (OR = 0.95; 95% CI: 0.86–1.06) [86]. We performed a meta-analysis of the two aforementioned studies including three sets of case–control data using the OR and 95% CI values with R version 4.0.2. We evaluated the quality of the included studies according to the Newcastle Ottawa scale (NOS) [90]. The results showed that the quality of the included studies was good (Supplementary Table S1). Heterogeneity among the three sets of included research data was not negligible (*p* = 0.13, *I*^2^ = 52.0%). For the three cohort studies, the *p*-values obtained from the Egger’s test and Begg’s test of publication bias analysis were 0.602 and 0.195, respectively, which were greater than 0.10, indicating that there was no significant publication bias. Therefore, we utilized the combined OR (0.85) and 95% CI (0.74–0.98) with a random effect model. The 95% CI does not contain 1, indicating that antidepressant use reduces the risk of gliomas (Figure 1). Our meta-analysis had some limitations. First, the included studies were few. Second, the studies involved a small sample size. Third, there was significant heterogeneity. One reason that heterogeneity cannot be ignored may be that the diagnosis of depression is related to the heterogeneity of the disease itself [91,92]. An emerging concept is that mood disorders may exist on a continuous spectrum. Genetic and biological studies have confirmed that mood disorders and mental disorders share a common biological basis, namely that these disorders are not discrete categories and may form part of a continuum or spectrum of mood disorders [91,92]. Physicians may encounter patients who meet more than one diagnostic criterion at a time or different criteria at different times, and the known differential diagnosis can be quite imprecise, depending largely on timing, progression, and overlap between emotional and psychotic symptoms [91,92]. Therefore, the continuum spectrum of mood disorders is important for diagnosis and treatment, which may also account for the high heterogeneity in the diagnosis of depression by clinicians [91,92]. Therefore, these results have limited generalizability.

A study has shown that the prognosis of patients with gliomas did not worsen without SSRI compared to with (hazard ratio [HR] = 1.50; 95% CI: 1.00–2.42) [87]. In addition, results from two other cohort studies also showed that TCAs (HR = 0.83; 95% CI: 0.53–1.30) [88] and SSRI (HR = 1.27; 95% CI: 0.98–1.64) [89] use did not affect the risk of death in patients with gliomas. Therefore, the above three studies suggest that the use of antidepressants dose not affect the prognosis of patients with gliomas.

Regarding the mechanism of action, antidepressants are related to the occurrence and development of gliomas. Clomipramine, a TCA, synergistically induces apoptosis in glioma cells with dexamethasone [93]. Animal experiments have shown that ticlopidine can enhance the ability of imipramine, a TCA, to induce autophagy-related cell death and improve the survival rate in glioma mice [94]. Fluoxetine, a SSRI, can also repair brain cell damage and protect hippocampal neurons [95]. In fact, glioma and depression have been shown to share the same pathophysiological molecular pathways, which may influence the choice of drug therapy for clinicians and patient outcomes [96,97]. The exact mechanism by which depression develops is unknown but appears to be a combination of several neurotransmitter disorders, including glutamate, GABA, corticotrophin-releasing hormone (CRN), neuropeptide Y, norepinephrine, and dopamine [96]. In gliomas, histamine has been found to cause hyperpolarization of cell membranes, serotonin has been found to promote cell growth, norepinephrine inhibits glucose uptake, and dopamine is associated with glioma proliferation [96]. Therefore, between glioma and depression, serotonin, norepinephrine, and dopamine appear to be involved in the disease process [96]. In addition, overexpressed receptors in gliomas are associated with depression [96,97]. The serotonin receptor subgroup, 5-HT2cR, was increased in interferon-treated glioma cells, and both diseases, particularly depression, are associated with serotonin receptors, suggesting a common signaling pathway between the two diseases [96,97]. In depression, decreases in calcium-binding protein P11 (a 5-HT receptor-associated signaling molecule) and brain-derived neurotrophic factor (BDNF) have been demonstrated. BDNF can inhibit the growth of gliomas and induce cell apoptosis [96,98]. Given the shared receptors, it is tempting to assume that signaling in one disease might influence the progress of another—either glioma’s effect on depression or depression’s effect on depression. Further research is needed to confirm whether glioma and depression do indeed share this common signaling receptor, and whether it is involved in the development of one pathology or induces the progression of the other.

There is no international guideline to guide clinicians regarding antidepressant use in patients with gliomas, and the efficacy and safety of antidepressants are uncertain. Larger and more rigorous prospective studies are required to evaluate the impact of antidepressants on the occurrence and prognosis of gliomas, explore the safety of the clinical use of antidepressants and repositioning of drugs, and provide new treatment options for patients with gliomas.

## 12. Conclusions

Patients with gliomas have a higher incidence of depression after diagnosis. Clinicians should screen the psychological status of patients with gliomas. Most studies of depression in patients with gliomas are small, cross-sectional, or retrospective, and there is still a lack of large, prospective studies to explore the risk factors of depression in patients with gliomas. There is no evidence that glioma is a risk factor for depression. The QoL of patients with gliomas can be directly reduced by depression. Psychological and cognitive interventions can improve the QoL of patients with gliomas. Antidepressant use may be a treatment strategy for patients with gliomas and comorbid depression. However, due to the heterogeneity of depression diagnosis, it is difficult for patients with gliomas to complete the survey assessment of QoL, and the results of previous studies are largely not reliable. In order to study the effects of depression and antidepressants on the occurrence and prognosis of gliomas, and provide clinicians and oncologists with new treatment strategies to improve the prognosis of patients with gliomas, more rigorous and comprehensive research is needed.

## Figures and Tables

**Table 1:** Prevalence of mental disorders and depression in glioma.

Author	No. of Cases (Glioma)	Mental Disorders ^a^ (%)	Depression Assessment Method	Major Depression ^b^ (%)	Minor Depression (%)	Relationship between Depression and Prognosis in Patients with Gliomas
Song et al. [20]	71	53.5%/70.4%/32.4% (depression/anxiety/cognitive impairment)	HAMD	53.3%	Not mentioned	Not mentioned
Bunevicius et al. [21]	63	28%/36% (depression/anxiety)	HADS	14% (high-grade glioma (*n* = 43))/11% (low-grade glioma (*n* = 20))	19% (high-grade glioma (*n* = 43))/ 26% (low-grade glioma (*n* = 20))	No correlation between depression and the overall survival (OS) of low-grade or high-grade glioma patients (*p* = 0.12 and *p* = 0.55, respectively)
Hao et al. [22]	190	28.4%-32.6%/36.3% (depression/anxiety)	HADS and SDS	6.3–15.2%	17.4–22.1%	Depression diagnosed by SDS was associated with a shorter OS (*p* = 0.016), while depression diagnosed by HADS was not associated with a shorter OS (*p* = 0.086)
Wang et al. [23]	249	46% (depression)	HADS	Not mentioned	Not mentioned	Depression may correlate with QoL and outcomes of patients ^c^
Piil et al. [24]	30	26.7% (depression)	HADS	Not mentioned	Not mentioned	Not mentioned
Rooney et al. [13]	155	20.6% ± 6.4% (major depressive disorder)	HAD-D	20.6% ± 6.4%	Not mentioned	Not mentioned
Kilbride et al. [25]	51	13–22%	HADS	6%	Not mentioned	Not mentioned

HAMD, Hamilton Depression Scale; HADS, Hospital Anxiety and Depression Scale; SDS, Self-rating Depression Scale; OS, overall survival; QoL, quality of life; HAD-D, Depression Scale (Depression subscale). ^a^ including anxiety and depression and so on. ^b^ including moderate depression and severe depression. ^c^ this is the conclusion of the article [23], without providing data directly related to survival or death.

**Table 2:** Characteristics of articles on the relationship between antidepressants and glioma.

Author	Year	Country	Antidepressants	OR (95% CI) (Adjusted)/HR (95% CI) (Adjusted) ^a^	Name of Drugs or Source of Drugs
Walker AJ [85]	2011	UK	TCAs	0.59 (0.42–0.81)	Section 4.3.3 of the British National Formulary (BNF)
Pottegård A [86]	2016	Denmark	TCAs/SSRI	TCAs: 0.89 (0.75–1.06) SSRI: 0.95 (0.86–1.06)	No specific description
Caudill JS [87]	2011	America	SSRI	0.65 (0.41–0.99)	Escitalopram, Fluoxetine, Fluvoxamine, Paroxetine, or Sertraline
Walker AJ [88]	2012	UK	TCAs	0.83 (0.53–1.30)	Section 4.3.3 of the British National Formulary (BNF)
Otto-Meyer S [89]	2019	America	SSRI	1.27 (0.98–1.64)	Escitalopram, Fluoxetine, Fluvoxamine, Paroxetine, or Sertraline

SSRI, selective serotonin reuptake inhibitor; TCAs, tricyclic antidepressants; OR, odds ratio; HR, hazard ratio; CI, confidence interval. ^a^ Refs. [85,86] are case–control studies, and case–control studies correspond to OR values; Refs. [87,88,89] are cohort studies, and cohort studies correspond to HR values.

## Data Availability

The authors confirm that all data underlying the findings are fully available without restriction. All data are included within the manuscript.

## Supplementary Materials

The following supporting information can be downloaded at: https://www.mdpi.com/article/10.3390/jcm11164811/s1, Table S1: Methodology and reporting assessment of the enrolled of the enrolled case-control studies [85,86].
