# Supplementary material for: Depression and Quality of Life in Patients with Gliomas: A Narrative Review
Source: J Clin Med. 2022 Aug 17;11(16):4811. doi: 10.3390/jcm11164811 (PMC9410515; doi:10.3390/jcm11164811)
Supplement: Supplementary file 1 [file jcm-11-04811-s001.zip › jcm-1817322-supplementary.pdf]

Supplementary Table S1. Methodology and reporting assessment of the enrolled of the enrolled case-control studies.

Studies	Selection				Comparability	Exposure factor measurement			Score
	Case appropriate	Case representative	Selection of control	Determination of control		Determination of exposure factors	Same method to determine exposure factors	No response rate	
Walker AJ [85]	Y	Y	Y	Y	N, N	Y	Y	Y	7
Pottegård A [86]	Y	Y	Y	Y	N, N	Y	Y	Y	7

Y, criteria satisfied; N, criteria not satisfied.
